# Facial Nerve Preservation in Advanced Facial Cutaneous Squamous Cell Carcinoma With Parotid Invasion: A Case Report

**DOI:** 10.7759/cureus.111712

**Published:** 2026-06-29

**Authors:** Camila V Gonçalves, Antonio V Priante, Leticia G Da Cunha Bueno

**Affiliations:** 1 Department of Medicine, Universidade De Taubaté, Taubaté, BRA

**Keywords:** head and neck oncology, skin neoplasms, squamous cell carcinoma, supraclavicular flap, surgery

## Abstract

To report the management of an advanced cutaneous squamous cell carcinoma (SCC) with parotid gland invasion, emphasizing surgical treatment with facial nerve preservation and reconstruction using a supraclavicular fasciocutaneous flap.

A 64-year-old man presented with a rapidly growing ulcerated lesion in the right malar region measuring approximately 5.0 cm × 4.0 cm. Imaging suggested parotid gland involvement without regional lymph node metastasis (cT3N0M0), which was subsequently confirmed histopathologically. The patient underwent wide local excision and parotidectomy extending to the deep lobe following identification of a positive deep margin on frozen-section analysis, while preserving the facial nerve. Reconstruction was performed using a supraclavicular fasciocutaneous flap. Histopathological examination confirmed a moderately differentiated SCC with parotid gland invasion and clear surgical margins. Adjuvant radiotherapy was subsequently administered.

Advanced cutaneous SCC of the face requires multidisciplinary management. Radical surgical resection with intraoperative margin assessment may allow preservation of critical structures such as the facial nerve, while the supraclavicular fasciocutaneous flap remains a reliable reconstructive option for extensive cervicofacial defects.

## Introduction

Skin cancer is the most common malignant tumor in humans, with basal cell carcinoma, squamous cell carcinoma (SCC), and melanoma being the most frequent types. SCC accounts for approximately 20% of cases [1]. It originates from the proliferation of atypical squamous cells that constitute the most superficial layers of the skin and is associated with chronic ultraviolet radiation exposure, advanced age, and immunosuppression [2].

The incidence of SCC has been increasing worldwide, particularly among the Caucasian population [3]. For non-melanoma skin cancer in Brazil, the estimated risk for each year from 2026 to 2028 is 130.42 new cases per 100000 men and 115.75 per 100000 women [4].

SCC primarily affects sun-exposed areas such as the scalp, face, and neck [2]. However, it is not limited to these sites and may develop on any part of the body. Patients commonly present with erythematous lesions resembling traumatic wounds or non-healing, scaly lesions, often associated with bleeding. The tumor may show rapid growth and frequently presents with ulceration. In some cases, it may mimic warts or traumatic lesions, which contributes to delayed diagnosis and complicates treatment, often requiring more complex procedures such as mutilating surgeries with resection of adjacent structures, lymphadenectomy, more elaborate reconstructions, and adjuvant radiotherapy [2,4,5].

Specifically in the facial region, parotid gland invasion by cutaneous SCC represents locally advanced disease and is associated with higher tumor stage, poorer oncologic outcomes, and greater surgical complexity. Furthermore, the close anatomical relationship between the parotid gland and the facial nerve makes preservation of neural function a key consideration during surgical treatment, given its substantial impact on postoperative functional outcomes and quality of life [5-7].

Adequate clinical suspicion and biopsy of these lesions allow for accurate and early diagnosis, which is essential for implementing appropriate treatment and achieving the best possible outcomes [5,6].

This case highlights the management of an advanced facial cutaneous SCC with parotid gland invasion, illustrating that radical oncologic resection with intraoperative margin control can be safely performed while preserving the facial nerve. It also underscores the supraclavicular fasciocutaneous flap as a reliable, low-morbidity pedicled reconstructive option for extensive cervicofacial defects, particularly in patients with comorbidities or in settings where microsurgical reconstruction is not available. The report reinforces the importance of multidisciplinary planning to achieve adequate oncologic control while maintaining functional and aesthetic outcomes in advanced skin cancer.

## Case presentation

A 64-year-old male, a retired gravedigger with a history of chronic sun exposure, sought medical care reporting the appearance of an ulcerated lesion in the right malar region for six months, with rapid and progressive growth, associated with episodes of pain and local bleeding. His medical history was significant for hypertension and diabetes mellitus, and he was a former smoker and alcohol user.

The patient had Fitzpatrick skin phototype II, with actinic keratoses and signs of photodamage on the skin of the face, neck, and upper limbs. In the right preauricular and malar region, an ulcerated and infiltrative lesion with elevated and irregular borders was observed. The lesion bled on palpation, measured 5.0 cm × 4.0 cm, and was fixed to the deep planes (Figure 1). No lymphadenopathy was detected in the face or neck.

**Figure 1:**
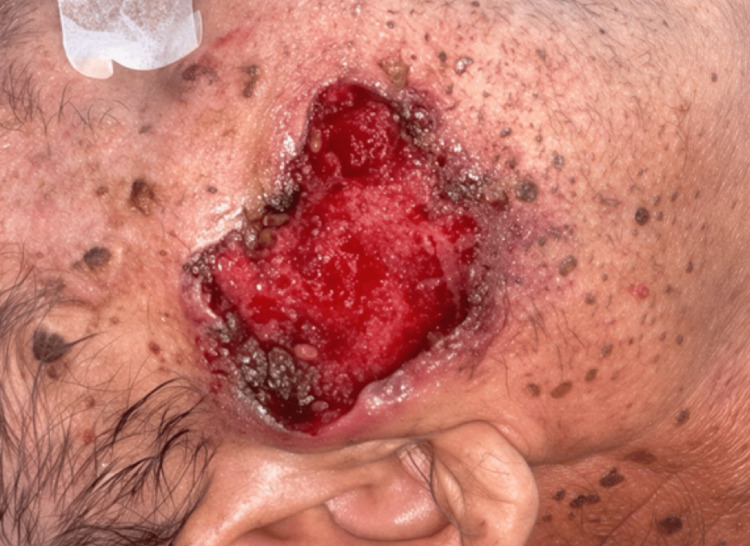
Clinical appearance at initial evaluation

The diagnostic hypothesis of cutaneous SCC was confirmed by incisional biopsy. Following the diagnosis, tumor staging was completed with computed tomography of the neck, face, and chest, which demonstrated a lesion measuring 5.3 cm × 4.6 cm in its largest dimensions, with a thickness of 0.9 cm, and no clear cleavage plane between the tumor and the parotid gland (Figure 2). No lymphadenopathy or pulmonary nodules were identified. The tumor was classified as T3N0M0, clinical stage III [7]. Preoperative evaluation revealed no significant abnormalities, and surgical treatment was therefore proposed.

**Figure 2:**
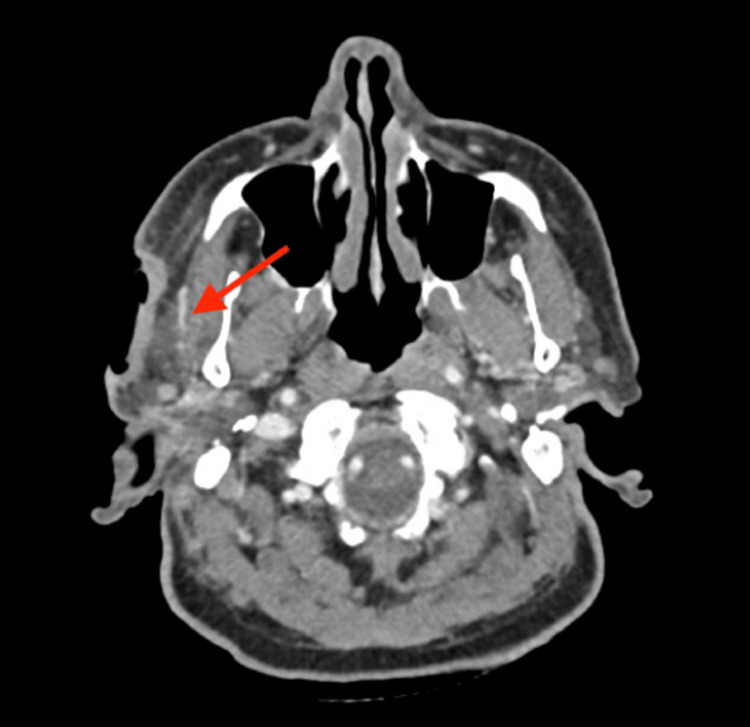
Axial contrast-enhanced CT scan of the head and neck. The red arrow indicates suspected tumor infiltration of the right parotid gland CT, computed tomography

The patient underwent surgical excision of the lesion followed by reconstruction with a supraclavicular flap. Lateral safety margins of approximately 1.0 cm were obtained, and the deep margin included the superficial portion of the parotid gland. Prior to resection, the ulcerated tumor surface was protected with a surgical gauze dressing sutured to the surrounding skin. During parotid resection, the main trunk of the facial nerve and its branches were identified and preserved (Figure 3).

**Figure 3:**
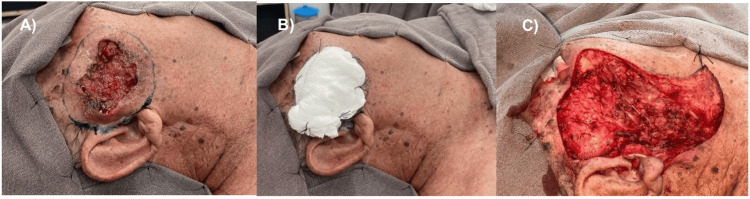
A) Delineation of the lateral margins. (B) Protection of the tumor surface. (C) Post-resection view demonstrating the facial nerve trunk and its branches

After specimen removal, intraoperative frozen-section analysis of the surgical margins was performed. The lateral margins were negative for neoplasia; however, the deep margin (parotid tissue) was involved. Based on this finding, resection of the deep lobe of the parotid gland was carried out, and a new frozen-section analysis of the deep margin confirmed the absence of neoplasia. Reconstruction was performed using a supraclavicular fasciocutaneous flap (Figure 4).

**Figure 4:**
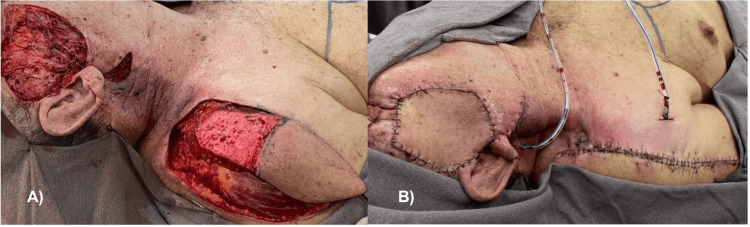
(A) Elevation of the supraclavicular flap. (B) Flap reconstruction of the surgical defect and closure of the donor site

The patient had an uneventful postoperative course, with no complications and no impairment of facial expression. Histopathological examination of the surgical specimen revealed a moderately differentiated SCC with a keratinizing architectural pattern, measuring 6.0 cm × 3.0 cm in its largest dimensions and with a depth of invasion of 0.6 cm. No lymphovascular or perineural invasion was identified. All surgical margins were negative, with the closest margin being the deep margin (2.0 mm); all lateral margins were greater than 10 mm. No intraparotid lymph nodes were identified.

Following multidisciplinary tumor board discussion, adjuvant conformal radiotherapy was administered at a total dose of 50 Gy in daily fractions of 2.0 Gy. During the course of radiotherapy, the patient developed mild radiodermatitis. At 23 months of follow-up, the patient remains disease-free, with no signs of recurrence (Figure 5).

**Figure 5:**
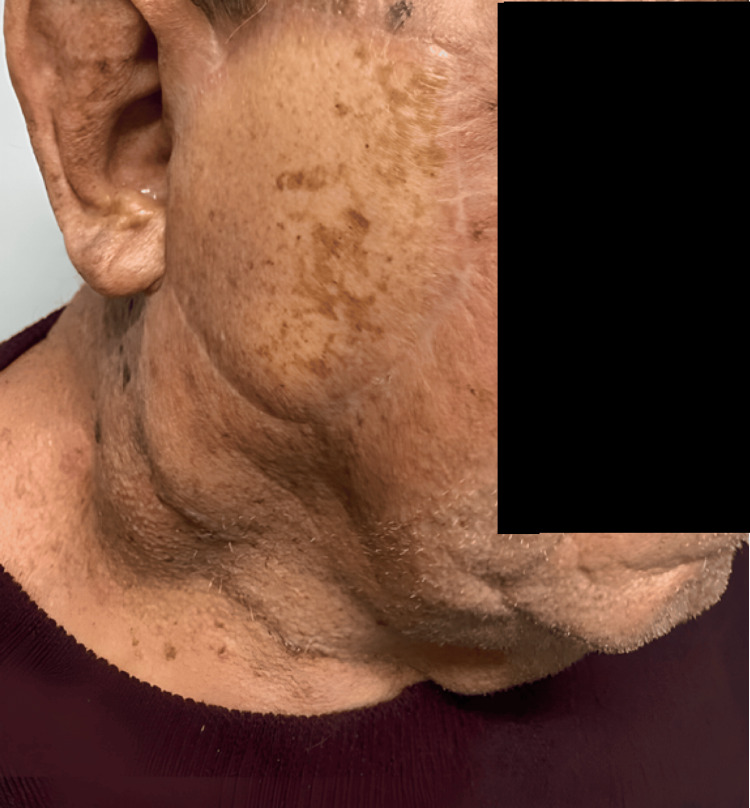
Postoperative appearance at the completion of radiotherapy

## Discussion

Cutaneous SCC is one of the most frequent malignant skin tumors worldwide. Although most lesions are diagnosed at early stages and treated with local excision, a subset of patients presents with locally advanced disease involving deeper anatomical structures such as the parotid gland. These cases require complex surgical management and multidisciplinary treatment strategies. According to a recent study, there has been a considerable increase in the number of advanced non-melanoma skin cancer cases with metastasis following the COVID-19 pandemic, due to the interruption of medical services in 2020 and a reduction in patients seeking medical care during that period [5].

For patients diagnosed with SCC, prognosis worsens substantially in the presence of high-risk features, regional nodal involvement, unresectable disease, or distant metastasis [6]. Diagnosis of the disease at advanced stages remains a frequent scenario, resulting in high morbidity and mortality rates [6,7]. Delayed access to appropriate care directly affects prognosis, as early diagnosis represents the best approach to preventing disease progression, improving quality of life, reducing the need for more invasive treatments, and increasing patient survival and the likelihood of cure [5-7].

There are several treatment modalities for cutaneous SCC, including surgery, radiotherapy, immunotherapy, targeted therapy, and chemotherapy, which may be used alone or in combination depending on tumor stage, patient clinical condition, and tumor location. Particularly in advanced tumors, a multidisciplinary approach is required [2,8]. Most primary tumors are localized and low-risk and can be treated with outpatient surgery under local anesthesia [2].

In patients with high-risk cutaneous SCC, Mohs micrographic surgery is a recommended approach to achieve local disease control. This technique involves lesion removal with meticulous assessment of both peripheral and deep margins using intraoperative frozen-section analysis. Studies have demonstrated its effectiveness in the treatment of primary cutaneous SCC, with reported local recurrence rates ranging from 1.2% to 4.1%, as well as reduced rates of nodal metastasis and disease-related mortality [9]. More extensive cases that require resection of deeper tissues often necessitate complex reconstruction of the resulting defect [10,11].

Free flaps have become the mainstay for reconstruction of large defects in the head and neck region. However, due to prolonged operative time and the typical profile of oncologic patients, who are often elderly, smokers, and have multiple comorbidities, not all patients are suitable candidates for microvascular free-flap reconstruction. Additionally, in many settings, the availability of trained teams capable of performing microsurgical reconstruction remains limited. Consequently, pedicled flaps continue to represent a valuable alternative. Their faster dissection and transfer reduce the morbidity associated with prolonged general anesthesia and may eliminate the need for intensive postoperative care [11].

In our patient, a supraclavicular flap was selected. This flap represents a versatile option for cervicofacial reconstruction, as the shoulder region and its adjacent areas offer skin with color and texture similar to those of the face and neck. Furthermore, it is supplied by a pedicle that is easy to dissect, allowing its broad application in the reconstruction of cutaneous SCC defects of the head and neck [10].

It is important to emphasize that reconstruction of exposed and mobile areas, such as the face, is essential for patient social interaction. Beyond aesthetic considerations, reconstruction should aim to preserve mechanical function and appropriate facial animation. Adequate coverage of major vessels, bones, and nerves is also crucial to enable adjuvant radiotherapy when indicated [10,12].

When high-risk features for recurrence are identified after surgery, such as positive or close margins, perineural invasion, invasion extending beyond the skin, or the presence of nodal metastases, adjuvant radiotherapy is recommended [8].

In cases in which the patient does not meet the criteria for surgical treatment, radiotherapy to the primary tumor may be considered as an exclusive therapeutic option or in combination with systemic therapies, including immunotherapy, chemotherapy, or targeted therapies [2,8].

In our patient, due to the extent of the tumor, surgery was performed under general anesthesia, with an indication for resection of deep tissues, including the parotid gland. Intraoperative frozen-section analysis was employed, allowing identification of the need for extension of the deep margin during surgery. As there was no infiltration of the facial nerve or its branches, nerve preservation was feasible. Following surgical treatment, the patient underwent adjuvant radiotherapy.

Recurrence of SCC most commonly occurs within two years after diagnosis. Therefore, close and continuous clinical surveillance is recommended. In addition to regular medical consultations and examinations, patients are encouraged to adopt strict photoprotection measures [2]. At present, our patient remains under follow-up with the dermatology and head and neck surgery teams, with no clinical signs of disease recurrence.

## Conclusions

Due to population aging and the increasing incidence of exogenous immunosuppression, the number of skin cancer cases is expected to continue rising, despite advances in staging and disease management technologies. Advanced skin cancer requires complex, multidisciplinary treatment, including surgical resection, often associated with lymphadenectomy, extension to adjacent structures, reconstruction, and adjuvant therapies such as radiotherapy.

Especially for patients with cutaneous SCC invading the parotid gland without clinical or intraoperative evidence of facial nerve invasion, oncologic resection with facial nerve preservation may be possible when guided by careful surgical planning and margin assessment. Early diagnosis remains fundamental for improving cure rates and patient quality of life, including functional and aesthetic outcomes. After treatment, close follow-up is necessary because the risk of recurrence remains significant in advanced cutaneous SCC.

## References

[1] Stern RS (2010). Prevalence of a history of skin cancer in 2007: results of an incidence-based model. Arch Dermatol.

[2] Wysong A (2023). Squamous-cell carcinoma of the skin. N Engl J Med.

[3] Brougham ND, Tan ST (2014). The incidence and risk factors of metastasis for cutaneous squamous cell carcinoma-implications on the T-classification system. J Surg Oncol.

[4] Instituto Nacional de Câncer (INCA (2026). Instituto Nacional de Câncer (INCA): estimativa (Portuguese). Estimativa 2023: Incidência de câncer no Brasil; INCA: Brasília, Brazil.

[5] Taccioli F, Blessent CG, Paganelli A (2024). Delay in cutaneous squamous cell carcinoma diagnosis due to interrupted services is associated with worse prognoses and modified surgical approaches. Cancers (Basel).

[6] 6. Perez M, Abisaad JA, Rojas KD, Marchetti MA, Jaimes N (2026). Skin cancer: primary, secondary, and tertiary prevention. Part I. J Am Acad Dermatol.

[7] Brierley JD, Gospodarowicz MK, Wittekind C (2017). Carcinoma of skin (excluding eyelid, head and neck, perianal, vulva, and penis). TNM Online.

[8] Stratigos AJ, Garbe C, Dessinioti C (2020). European interdisciplinary guideline on invasive squamous cell carcinoma of the skin: Part 2. Treatment. Eur J Cancer.

[9] Tschetter AJ, Campoli MR, Zitelli JA, Brodland DG (2020). Long-term clinical outcomes of patients with invasive cutaneous squamous cell carcinoma treated with Mohs micrographic surgery: a 5-year, multicenter, prospective cohort study. J Am Acad Dermatol.

[10] Hamidian Jahromi A, Horen SR, Miller EJ, Konofaos P (2022). A comprehensive review on the supraclavicular flap for head and neck reconstruction. Ann Plast Surg.

[11] Takahashi GG, Américo D, Figueiredo P (2023). Supraclavicular flap for head and neck oncological reconstruction: a series of 62 cases. Rev Bras Cir Plást.

[12] Muzic JG, Schmitt AR, Wright AC (2017). Incidence and trends of basal cell carcinoma and cutaneous squamous cell carcinoma: a population-based study in Olmsted County, Minnesota, 2000 to 2010. Mayo Clin Proc.

